# Supplementary material for: Content-rich biological network constructed by mining PubMed abstracts
Source: BMC Bioinformatics. 2004 Oct 8;5:147. doi: 10.1186/1471-2105-5-147 (PMC528731; doi:10.1186/1471-2105-5-147)
Supplement: Additional File 2 — The original results of the above study (non-essential files are deleted to keep the file size under the limit set by BMC bioinformatics). [file 1471-2105-5-147-S2.bz2 › chilibotAdditionalFile2/dip40/466ID10556181E10494/html/CCR4_CXCL12.html]

 


 **CCR4** and **CXCL12** 
  
Found 65 abstracts in PubMed, retrieved 40.  
 

 What does Google say? 
 PDF only 
| .edu only 

---

**Interactive relationship** (e.g. stimulation, inhibition, etc)

**Neutral relationship**- Stromal cell derived factor 1  [ **CXCL12** ]  CXC chemokine receptor 4  [ **CCR4** ]  interactions play a central role in CD4 T cell accumulation in rheumatoid arthritis synovium.  Ref: 11086103 J Immunol, 2000
- Previous reports have shown that the Gi protein coupled CXC chemokine receptor 4  [ **CCR4** ]  is activated by stromal cell derived factor 1  [ **CXCL12** ]  SDF 1  [ **CXCL12** ] .  Ref: 11069075 Eur J Immunol, 2000
- Stimulation of matrix metalloprotease 3 release from human chondrocytes by the interaction of stromal cell derived factor 1  [ **CXCL12** ]  and CXC chemokine receptor 4  [ **CCR4** ] .  Ref: 11817585 Arthritis Rheum, 2002
- The chemokine stromal cell derived factor 1  [ **CXCL12** ]  SDF 1  [ **CXCL12** ]  is the natural ligand for CXC chemokine receptor 4  [ **CCR4** ]  CXCR4.  Ref: 11352904 J Biol Chem, 2001
- We report that stromal cell derived factor SDF 1  [ **CXCL12** ]  has the remarkable capacity to induce sustained signaling through CXC chemokine receptor 4  [ **CCR4** ]  CXCR4.  Ref: 10934220 J Exp Med, 2000
- The human CXC chemokine receptor 4  [ **CCR4** ]  CXCR4 is a receptor for the chemokine stromal cell derived factor SDF  [ **CXCL12** ]  1alpha and a co receptor for the entry of specific strains of human immunodeficiency virus type I HIV 1.  Ref: 11551942 J Biol Chem, 2001
- A simplified model of the binding interaction between stromal cell derived factor 1  [ **CXCL12** ]  chemokine and CXC chemokine receptor 4  [ **CCR4** ] .  Ref: 12678810 Protein Pept Lett, 2003
- We could show that SDF  [ **CXCL12** ]  1alpha exposure led to a down regulation of the gene expression of the chemokine receptors  **CCR4** , CXCR4, and CXCR5, which are associated with cell motility and podia formation, indicating a negative feedback control.  Ref: 11867680 J Leukoc Biol, 2002
- Three chemokines in particular,  **CXCL12**  SDF 1  [ **CXCL12** ] , CCL17 TARC, and CCL22 MDC, recently have been shown to be strong and rapid activators of platelet aggregation and adhesion after their binding to platelet CXCR4 or  **CCR4** , when acting in combination with low levels of primary agonists.  Ref: 12851650 Microcirculation, 2003
- Fractalkine, SDF  [ **CXCL12** ]  1alpha, RANTES and MDC inhibited the I Ba in CX3CR1, CXCR4, CCR5 and  **CCR4**  expressing G1A1 cells, respectively.  Ref: 11880151 J Neuroimmunol, 2002

**Non-interactive relationship** (e.g. studied together, co-existance, homology, etc.)

- Several lines of evidence have suggested that a CXC chemokine receptor 4  [ **CCR4** ]  CXCR4 stromal cell derived factor 1  [ **CXCL12** ]  SDF 1  [ **CXCL12** ] .  Ref: 11927659 J Leukoc Biol, 2002
- The most active chemokines for resting Th IL 10, in terms of calcium mobilization and in vitro migration, were in order of potency CCL2 monocyte chemoattractant protein 1, CCR2 ligand, CCL4 macrophage inflammatory protein 1beta, CCR5 ligand, CCL3 macrophage inflammatory protein 1alpha, CCR1 5 ligand, CCL17 thymus and activation regulated chemokine,  **CCR4**  ligand, CCL1 I 309, CCR8 ligand,  **CXCL12**  stromal derived factor 1, CXCR4, and CCL11 eotaxin, CCR3 ligand.  Ref: 11145678 J Immunol, 2001
- The Stromal Derived Factor 1  **CXCL12**  CXC Chemokine Receptor 4  [ **CCR4** ]  Biological Axis in Non Small Cell Lung Cancer Metastases.  Ref: 12626353 Am J Respir Crit Care Med, 2003
- In late angiogenesis, stromal cell derived factor SDF 1  [ **CXCL12** ]  and its receptor CXC chemokine receptor 4  [ **CCR4** ]  CXCR4 act in an organ specific manner to promote the formation and development of large blood vessels supplying the gastrointestinal tract.  Ref: 12193143 Clin Sci Lond , 2002
- Among the chemokine receptors expressed on platelets, the CXCR4 is the receptor for chemokine stromal cell derived factor 1  [ **CXCL12** ] , and the  **CCR4**  is the receptor for macrophage derived chemokine.  Ref: 10891429 Blood, 2000
- A chemokine, stromal cell derived factor pre B cell growth stimulating factor  [ **CXCL12** ]  SDF 1  [ **CXCL12** ]  PBSF  [ **CXCL12** ] , and its primary physiologic receptor, CXC chemokine receptor 4  [ **CCR4** ]  CXCR4, are essential for B lymphocyte production and colonization of bone marrow by myeloid lineage progenitors during embryogenesis.  Ref: 11197205 Int J Hematol, 2000
- A chemokine, SDF 1  [ **CXCL12** ]  PBSF  [ **CXCL12** ] , and its receptor, CXC chemokine receptor 4  [ **CCR4** ] , as mediators of hematopoiesis.  Ref: 11197205 Int J Hematol, 2000
- METHODS Expression of the SDF 1  [ **CXCL12** ]  receptor CXC chemokine receptor 4  [ **CCR4** ]  CXCR4 on mast cells was examined by RT PCR and flow cytometry.  Ref: 11306951 Int Arch Allergy Immunol< MedlineTA>Int Arch Allergy Immunol, 2000
- We show that both CXC chemokine receptor 4  [ **CCR4** ]  CXCR4 and SDF 1  [ **CXCL12** ]  mRNA are expressed in several human glioblastoma multiforme tumor tissues and in two human glioblastoma cell lines, U87 MG and DBTRG 05MG.  Ref: 12702590 Cancer Res, 2003
- CXC chemokine receptor 4  [ **CCR4** ]  CXCR4, the high affinity receptor for stroma derived factor 1alpha SDF  [ **CXCL12** ]  1alpha, shows distinct patterns of expression in human CD34 haematopoietic progenitor cells induced to differentiate in vitro along the granulocytic and erythroid lineages.  Ref: 11122082 Br J Haematol, 2000
- We hypothesized that the CXC chemokine receptor 4  [ **CCR4** ]  stromal derived factor 1 axis may be involved in metastasis of CXCR4 tumor cells into the bone marrow and lymph nodes, which secrete the alpha chemokine SDF 1  [ **CXCL12** ] .  Ref: 12239174 Blood, 2002
- The chemoattractant stromal cell derived factor 1  [ **CXCL12** ]  SDF 1  [ **CXCL12** ]  and its receptor CXC chemokine receptor 4  [ **CCR4** ]  CXCR4 are key modulators of immune function.  Ref: 12122049 J Neurosci, 2002
- Here we identify the CXC chemokine receptor 4  [ **CCR4** ]  CXCR4 in interneuron precursors migrating from the basal forebrain to the neocortex and demonstrate that stromal cell derived factor 1  [ **CXCL12** ]  SDF 1  [ **CXCL12** ]  is a potent chemoattractant for isolated striatal precursors.  Ref: 12832536 J Neurosci, 2003
- In contrast to in vivo homing, both in vitro migration toward stromal derived factor SDF 1  [ **CXCL12** ]  and the average CXC chemokine receptor 4  [ **CCR4** ]  CXCR4 expression were lower in 5 FU treated cells.  Ref: 12829029 Exp Hematol, 2003
